# Table of Meteorological Conditions in the City of Buffalo for March, 1855

**Published:** 1855-05

**Authors:** Sanford B. Hunt


					﻿ART. V.—Record of Meteorological Conditions at Buffalo, for March,
1855. By Sanford B. Hunt, M. D.
o	h
O -	o
•	^—3	a
5	o	’?	.S	.2
Cd	7	C	*	Q	cd	c3
<1	So	m	xh^	"	g	£	g
. .	________00*02 O__________________________________g g g g £1 g g O g'5x £ OOP o o_
•aanjuiadoiaj, I	|S
fo aSarc.i 3S94Ba4f)|m o m	h w oq	io o co ko co co ct b- j< co ct b- t- co m mom b~ I12
•Xji I CO b- KO ■^’ba b~	OCT -7 i — Cl <0 O — O — ct ( . ,c -“ X D C. C b-
mmnTT	CT KO	CO	b-	CO-	CO	CO CO	CO	CO	CO	CD CT CO CO	CO	CO	b-	CO CO CO O O	00	’	CO
-piulllH U1.91VI TQ ao q,	J,	QQ	QQ	g; _oo CO	CO	00	00	OO 00 m CO	CO	CO	CT	CO CO Ci CT CT	CO	i ~	GO
>	r '*t"^O>»CJ0	00 COt'COCOt'CCOrtOKBCDtOt'lflHrfCCH
?UI°<I	CT CT	ct.	co	7)	co	oo	cc	i'	cc < - cc c.	-	c.	X'	c - i' ic -	-	cc	cc
5 -AiSd ubj[\-	ct CT o	cd	id	t-2	oo	ct cd	ct	o	ct'	ci cd cd 71	CT	ct	cd	id cd cd ct id	7i	oi	cd
Q ___________— CT CO CT CO CT CT______________________CTCOCOCOCOCOCTCTCTCTCTCTCTCTCTCTCTCOCO CT
I	CO CO CO	O CO CO CD	COCO COCO CO CO CO CO CO IO
<xunu I . .COCOCO	IO CO CO CO .	. .COCO .COCO .COCOCOCO . CD. co
-wattiaj; treapj|ci -4 ct ct o ct -4	cdodcdCTt4CTcdididcdb4b2od~-cibtodcdcd|CT
__r-H CO CO CO CT CO CO___________________<71 CO CO CO CO CO CO CT CT CT CT CT O' CO CT CT CT CO CT CO
I© OCT CTO KO	~ O O CO CT CT~ ct"4~ 75~cctdTcO ooCTooib-
• '.CainininTr I—~	cococoococococococooco co co co co co o
u	4!1 •	ulH|00 00 O O	00 00	OOOOCOOOOOOOOOOO	OOOOOt^jOO
2	Ico CO CO	COCO	CO CO CO co CO CO	CO	CO	CO CO	CO CO CO'CO co	co
H	'lUTO.T	Alan I” . CO ©	CO CO.	cococo . co co co	co	co	co .co	co co. co co co
o	f	. a.	D id co .o’ oo	cd cd	ct cd CT cd ct cd cd	-4	-4	ci id ct	cd cd ct o CT	b-
; t£  ______ I ct< CT CO CT CO CT	CO CO CO CT CO 7? CT CT CT “< CO CT CT CT CT CO CO CT
s ±-	.	■	-	-	IO
-• •amj.daiajr .	.	....	..............
Ph	’* o co co —< —. —<	NoomccinoftTfi^CTioio ci oi t- m ct- —<
__________ICT CO CO CO CT CO___________CO CO CO CO CO CT CT CT CT ICT CO CT CT CT CT CO CT CO
g g ^3 g . g g_________________________. g	. g g g
§ rgSo^fe <Z2 cz2 qq c/2	cd m z 02 z <5 zzaizz
E	p> r- ........... ......................................................
J ______________" — CC CC	17	7___________________________—'	CT CT CT CO CT KO CO —' CO CT CT —i	-J CT — CT
H spnoo jo j.ui y | —' o o i-h o o	oonoot-ocoooooo o co co o o '
PS	,0 O CTI GO	1—I	CO b-	O CT I - odd	<7C O CT ididodl CT KO lO IC	O KO CO O 100
W	. c,co ct co co CT o co ct coco co .co o co co cicococococococococomocob'
p,	Ajtpitunjj oooocioicoaoaiaoaico b- oo o ci ao ao ' cocciciciciccicncic'. oo oo oo m | oo
o	Ico COCOCO	CO COCO	COCO	CO CO	COCOCOCOCOCOCOCOCOCOCO CO I CT
S	•lino t Aian r? . co co co	co coco	coco	co co	cococococococccocococp . co j co
O	•a	U CT cd O CT b-‘	cd	CT 00 CO CT	l ~~ bt	CT	CO O CT	ci CO d d P b d C d hi Cl CT V |7"
. g,	_'CT	CT CO CO CO	CT	CO CO CT CO	CT CT	CT	CO CO CO	CTCTCTCTCOCTCTCOCTCTCTCOCO CO
S ►>	jj^,
pH	kz)
.	ct >d cd id o cd id id ct id b< ct ct cd cd id-4 cd ac ci CT o o’> cd 7? ook
ct   _	7’ CO CO CO —• CO CO — CO CO	COCO—HCOCOCOCO—’CTCTCOCOCOCOCOCOCT co_CT io [co
'!	8-a g d	Pj g:bg	g	. pt pt . . g '	g g
r=§oc2 <Z2<Z2CZ2CZ2<Z2CZ2	g 02 00 g 02 CQ CC CQ CO 02 W2 02 02 CO CZ2 <Z2
O P . . .	..	........
___________CO CT-CO CT CT CT	co CT id CT-CT CT CT CT ct —_CT CO CT —i h w CT CT ct__
spnoiojo^.cav oooooo__________________________________________________________co ooctooooooctoooctiqo
110 O o”ct CT~uO b-	O <0 7! CT CC O O	O 7) O CO CO CD lO CT O O CO
. -ifitimunu cocoocoCTiioco	cococtcocoocococoocoocococococooco ct
u	Ji	QO O CO 00	00	CO	CO O 00 O)	Cl	C	® O O 00	ffi O	OC	Cl Q Cl Cl	O	®	O)
O   __________>-H	—<	—'	Ct______
2	co	co co	cd	co	co co co	co	coTcb co	co	co	co co co co	co	oo
S	-iriTOT	Mazi	CT . ’-0	CO	CO	CO CO COCO	CO CD CO	CO	.	CO	CO CO CD. CO	.	CO	L.O
CT g	1	.	a.	•	D	xd	O o 00 cd	cd	CT	CT -4 b4 ci	cd	CT	CT ci I— b4	cd	a5	CT	bt CT CO CT	CT	CT	KO
<A Ml ____ CT CO CT CO CT CT	CT CO CO CT CO CO CT CT CT —J7! CT- CT CT CT CT 7? CO CO CT
J M	’H’
••17m /Tr»TZ>-r
-	n?< max odidoCT©~4bt	t4-d4'd'CTcdCTaiCT^i4'*cdo6b4cdb4cd'^4CTcdod
b~_______________Ct CO CO CT co CT	CT CO CT CO CO CO CT CT 7’ CT _CT — 71 CO_71_CT CT CO CO CT
S-aiL'®	.^.j
'Oi aicc ai czi	in vi cd ui r gq gq od gq ai cd co co (
Q r* .....................................................................................................    |
___________CO	CT CT COjCT CT CT	CTctcQctooctCTkqJoCTCTCT	ct _-h —'_ct CT ct	
spno[3jo;.axv =>	o o o o b- o_© S 2	5	5	2 ° 2 2	2	2iS	2 2 2° °S	I
• .	I io co n	co i— o o e	—<	I
-uioaaajo	UIki	cd ci cd ci cd	oi	ci cd ci cd ad oo	ci	ci	ci ci ci ci	ai	ci	ao ao cd cd © co	co
I <7?	O? C? CT? O? CJ	CJ	CQ	<7? OJ	CQ	Ol	<7> Ck 7^ 79	C9	O?	O1 O 7s? 7? C9	Gx
'	i	o o	o ^77	in	o
Q	i.7	—<	7 j i’	07 ('	me?
■upHJo’ui	in	7—i	cn c o	m o	—;
______________________ I	o	’	___________^3
________________________________________________________________________________W CT ID io ^CO^2Zi22222^S2^CTCTCTCTCTCTCTCTCT°S
*Snow with Nimbus all day. Cu.-St. moving S.W. in upper air.
Explanation of Signs used under the head of “Remarks."—The four
principal varieties of cloud, Nimbus, Stratus, Cirrus, and Cumulus, will be
recognized by their abbreviations. All forms of cloud may be reduced to
these elements, alone, or in combination. When in combination, they are
expressed by joining them with a hyphen, thus: Ci.-st., cirro-stratus; ci.-cu.,
cirro-cumulus; cu.-st., cumulus-stratus, etc., The four primitive forms repre-
sent the following kinds of clouds:
Nimbus, the common, uniform leaden-colorcd, rain-cloud.
Stratus, the horizontal, stratiform cloud, seen most frequently on the
horizon, and at morning.
Cirrus, the light, feathery, broken clouds of the upper air. Usually very
high and scattered.
Cumulus, the piled up form, occupying middle region of air, and looking
like great fleeces of wool. Seen most often in warm weather.
If under the head “remarks” only cne form of cloud is given, it refers to
the only kind seen that day. If two forms are given, the second refers to
the form of a second or third observation.
				

